#  Chain drugstores: Opportunities and Threats

**Published:** 2014

**Authors:** Mehdi Mohammadzadeh, Nazila Yousefi, Hesam Sharifinia


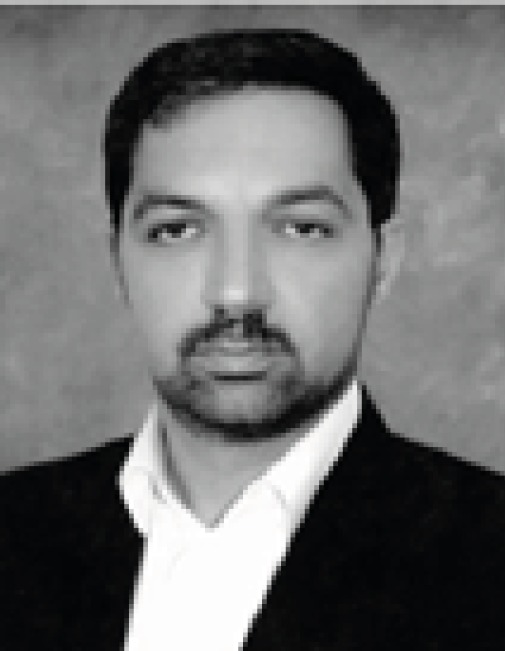


According to non-official data, despite official ban, over 50% of the pharmacies in Tehran are illegally managed by non pharmacist investors. It means despite the regulations ban, tendency to Chain pharmacy exist in Iran and some investor has more than 10 pharmacies. It means despite the regulations, tendency to Chain pharmacy exist. Benchmarking in other countries shows some advantages and disadvantages of chain pharmacies which if regulated properly could lead to patient benefits. Chain pharmacy regulations are wide varying all over the world. The organization structure might be based on the horizontal or vertical integration. In vertical integration as the pharmacy integrated with distributor, wholesaler, and factory, repeated mark ups are omitted and the unit costs could be lower. But due to the market dominance caused by this loop, it is not accepted in most of the countries. In horizontal integration chain pharmacy construct by merging some independent pharmacies and could be sole-ownership or shared-ownership. Shared owner CDS is collaboration of some independent pharmacies which managed by directorate board and directing manager. In sole-ownership CDS, board of directors is shareholders who assign a CEO. The chain pharmacy advantages from a positive perspective include increase in the number of pharmacies, better access, lower unit costs, extensive insurance contract, lower costs due to economies of scale, cost sharing, merging of pharmacies that their activities are not economically feasible, developing new services, Increase the internal quality, loyalty of customers, easier human resourcing, better management in financial crisis, better online facilities, integrated logistics, ability to use of logistic technologies such as RFID and GPS for optimal management of supply chain, and information sharing which not only has financial benefits for the pharmacy, prevents drug shortage by forecasting and planning. So allocate efficiency, quality, product diversity, and productivity improve in chain pharmacies. Although optimistically all these benefits could happen in the tight supervision and proper regulations, there are many disadvantages that must be taken into considerations. The disadvantages from the opponents› point of view include; decrease in a qualitative competition due to the decrease in the number of competitors and market dominance, prevention to enter other chain stores to creation monopoly, more limitation and less creativity for responsible pharmacists, loss of professional autonomy, uncertainty in the acceptance of legal responsibilities, limitation of job opportunities of the technical pharmacists to some limited number of chain pharmacies, less attention to health system goals as their main concern is reaching the their financial objectives and target turnover, Poor quality of services and less speed of service delivery. The most important concern of the opponents of chain pharmacies is decrease in drug availability, specifically in far places where activity of chain pharmacies is not feasible. Moreover, the problem of availability of the hard-to-find drugs will be escalated, since it is not economical to buy and store these sorts of drugs. Moreover, as the chain pharmacies are dependent upon big loans and their debt is more, in crisis, they would be the weakest part of the health chain. All above mentioned points could be summarized in to three major categories; “Accessibility”, “Quality” and “Economics”.

*Accessibility of the pharmacy*

Due to increase in the total number of pharmacies, accessibility increase. But it does not happen all over the country. Despite the number of pharmacies increase in the urban areas, rural and far-away areas face to drop in number of pharmacies. So in chain pharmacies› regulation, care should be exercised to avoid this problem by obligation of establishing chain in the rural areas (such as in Australian regulation).

*Accessibility to the prescription drugs*

In vertical integration due to the distributors› dominance, and in the horizontal integration due to the economical profits, high prescribed drugs are always available while rare drugs remain rare and it would be worse in the rural areas. 

*Drug accessibility*

In case of lack of proper regulation and supervision, due to the above mentioned reasons and tendency to sale non-medical items by higher profitability, drug accessibility would decrease.

*Personnel quality*

Powerful organization of Chain pharmacies enables them to employ high quality personnel. They can also could establish higher standards and train their personnel. However, in the lack of proper supervision, pharmacists are faced to problem for finding proper job and their professional creativity decrease. Empowerment of pharmacy owners and monopoly affect professional independence of pharmacists and fade roles.

*Service quality*

Some believe that lack of attention towards the prescription drugs and increase in the sales of non-prescription medicines and non-pharmaceutical products in spite of higher internal standards leads to poor quality of drug services. High load of work in chain pharmacies, lead to decrease in medical counseling; whereas, in independent pharmacies, due to a sole-ownership policy and fixed management quality of the services will sustained in the course of time. Meanwhile, some others believe that the quality of services in chain pharmacies is surely increased due to the economical power for employing more pharmacists by more payment. patient pay more attention to pharmacists because of the better services provided, and there is more inclination to paying counseling fee to the advising pharmacists compared to the independent pharmacies; counseling time also seems more comparatively. These different perspectives are because of the differences in the number of chain pharmacies and their authority over the market in different countries. 

*Contributing to health system’s objectives*

Although focusing on the profit may lead to the weakened role of pharmacist in the health care within the chain pharmacies and failure in achieving health system goals, by proper regulation and supervision, Chain pharmacies› facilities will be the opportunity to provide health services such as screening programs and training patients towards a better understanding of their health and their own potential needs.

*Pharmacy costs*

Due to the use of shared resources and economies of scale, costs are mostly reduced, though at times, high expenses in infrastructures or decoration may add up the total costs.

*Affecting the economy of independent pharmacies*

One can never deny the influence of chain pharmacies as a powerful competitor over the economy of independent pharmacies. In one view, integrating independent pharmacies with low turnover into chain pharmacies can save them; On the other hand, chain pharmacies can lead to more treat for independent pharmacies life.

*Patient costs*

It is expected to decrease Price of non-prescription and generic drugs and give a discount on generic drugs, which lead to decrease in the consumers’ drug expenses. But in many countries this is not the case. Although there is a more bargain power in chain pharmacies, the decrease in the consumer price is dependent upon the monitoring and controlling of pharmacy profit margin.

*Health section costs*

In chain pharmacies, due to the increase in the market power and monopoly, induced demand will increased, and because of the selling more non-prescription medicines and supplements, the general health system cost is increased.

## Conclusion

Due to the economical incentives, Chain pharmacies would not be avoidable and even though the legal limitation, they have been established illegally. As mentioned in this report, Chain pharmacies have some advantages and disadvantages and through a proper regulation and supervision, we can welcome its advantages and reduce disadvantages. Nevertheless, by keeping silent against this illegal phenomenon, society would bear disadvantages while being deprived of its possible advantages.

## Notes

*Mahdi Mohammadzadeh is currently working as assistant Professor of Pharmacoeconomic, School of Pharmacy, Shahid Beheshti University of Medical Sciences, Tehran, Iran. He could be reached at the following e-mail address: mohammadzadeh655@yahoo.com*

